# The Feasibility of Ultra-Sensitive Phonocardiography in Acute Chest Pain Patients of a Tertiary Care Emergency Department (ScorED Feasibility Study)

**DOI:** 10.3390/jpm12040631

**Published:** 2022-04-14

**Authors:** Sebastian Schnaubelt, Felix Eibensteiner, Julia Oppenauer, Andrea Kornfehl, Roman Brock, Laura Poschenreithner, Na Du, Enrico Baldi, Oliver Schlager, Alexander Niessner, Hans Domanovits, Dominik Roth, Patrick Sulzgruber

**Affiliations:** 1 Department of Emergency Medicine, Medical University of Vienna, 1090 Vienna, Austria; felix.eibensteiner@meduniwien.ac.at (F.E.); julia.oppenauer@meduniwien.ac.at (J.O.); andrea.kornfehl@meduniwien.ac.at (A.K.); roman.brock@meduniwien.ac.at (R.B.); laura.poschenreithner@meduniwien.ac.at (L.P.); na.du@meduniwien.ac.at (N.D.); hans.domanovits@meduniwien.ac.at (H.D.); dominik.roth@meduniwien.ac.at (D.R.); 2 Section of Cardiology, Department of Molecular Medicine, University of Pavia, 27100 Pavia, Italy; enrico.baldi@unipv.it; 3 Cardiac Intensive Care Unit, Arrhythmia, Electrophysiology and Experimental Cardiology, Fondazione IRCCS Policlinico San Matteo, 27100 Pavia, Italy; 4 Division of Angiology, Department of Internal Medicine II, Medical University of Vienna, 1090 Vienna, Austria; oliver.schlager@meduniwien.ac.at (O.S.); patrick.sulzgruber@meduniwien.ac.at (P.S.); 5 Division of Cardiology, Department of Internal Medicine II, Medical University of Vienna, 1090 Vienna, Austria; alexander.niessner@meduniwien.ac.at

**Keywords:** phonocardiography, diagnostics, chest pain, emergency medicine, emergency department

## Abstract

Background: Thoracic pain is one of the most frequent chief complaints at emergency departments (EDs). However, a respective workup in cases without clear electrocardiographic signs is complex. In addition, after having ruled out acute coronary syndrome (ACS), patients are often left with an unclear etiology of their symptoms. Ultra-sensitive phonocardiography is already used to rule out stable coronary artery disease (CAD); however, its feasibility in an ED-setting remains unknown. Methods: We prospectively used ultra-sensitive phonocardiography via the CADScor^®^System to measure hemodynamically stable patients with the chief complaint of chest pain during routine waiting times at a high-volume tertiary ED. Results: A total of 101 patients (49% male; 94% Caucasian; 61 (51–71) years; BMI 28.3 (24.2–31.6)) were enrolled. Patient workflow was not hindered, and no adverse events were recorded. In 80% of cases, a score was successfully calculated, with 74% at the first, 5% at the second, and 1% at the third attempt. Feasibility was judged as 9.0 (±1.8) by the patients, and 8.9 (±2.6) by the investigators on a 10-point Likert scale. Conclusions: Ultra-sensitive phonocardiography was found to be feasible in acute chest pain patients presenting to a tertiary ED. Thus, the CAD score measured during routine waiting times could potentially serve as an additional tool in a diagnostic pathway for thoracic pain.

## 1. Introduction

Thoracic pain is one of the most frequent chief complaints in emergency medicine [1,2]. While the majority of patients are eventually discharged with a non-cardiac/low-risk symptom etiology, acute coronary syndrome (ACS) must first be ruled out [2]. Currently, a combination of patients’ clinical presentation, electrocardiogram (ECG), and laboratory markers is recommended, potentially supplemented by scores [1]. Cases with normal or unspecific ECGs are complex, and often result in clinicians’ uncertainty on how to proceed [3]. Thus, novel tools for cardiovascular risk prediction in emergency departments (ED) and the following workup are needed. In this regard, surrogate markers for coronary artery disease (CAD) can be utilized: significant coronary artery stenosis generates turbulations in coronary blood flow, evident as intracoronary murmurs. Specific acoustic signal patterns combined with analysis techniques can identify these murmurs, and therefore hint towards existing CAD [4,5]. This approach using highly sensitive microphones, named phonocardiography, appears as non-invasive, inexpensive, and simple to perform [6,7]. We therefore aimed at assessing its feasibility in the setting of our tertiary-care, high-volume ED which provides comprehensive emergency and intensive care medicine [8,9]. Since phonocardiography has so far only been used and validated in stable CAD and in orderly and well-structured settings, such as outpatient clinics [10,11,12], providing data on its feasibility in busy and mostly overcrowded EDs [13] seems vital for further respective research endeavors: an ED not only differs from normal wards, outpatient departments, or even general physician offices in terms of time and resource management possibilities, there is also an increased physical and psychological strain on the personnel, further impacting on ED-reproducibility of measures that have previously been only assessed outside this unique environment [13,14,15,16,17].

## 2. Methods

### 2.1. Data Acquisition

Patients >40 years in stable cardiorespiratory condition presenting to the ED with the chief complaint of chest pain were screened.

Ultra-sensitive phonocardiography was prospectively performed using the CADScor^®^System (algorithm version 3.1, Acarix A/S, Denmark), a palm-sized portable device with an adhesive sensor applied to the patient’s chest (fourth left intercostal space) [7]. The measurement is performed via 3 min of recording with four breath holds for a reduction of acoustic interference. A numeric value regarding a continuum of risk is calculated (0–99 risk scale: low ≤20 points, intermediate 21–29, high ≥30) [7]. The score is calculated immediately after the recording using an integrated algorithm performing an analysis of the heart sounds together with age, gender, and blood pressure information. Combined with clinical risk evaluation, the CAD score shows good diagnostic accuracy for stable CAD detection [7,10], with a low-risk score (≤20) having a negative predictive value of up to 97%, thereby reliably ruling out stable CAD [7,10,11,12]. Further, it can perform a reclassification of intermediate risk patients to low risk [12].

Measurements were conducted during routine waiting times in a separate quiet room, a supine position, and after 5 min of rest. Results were blinded to patients and treating physicians or nurses. An overview of the measuring procedure can be found in Supplementary Table S1 [18].

### 2.2. Data Analysis

The primary study outcome was the utilization feasibility of the CADScor^®^System. The overall study population was stratified into subgroups concerning gender, CAD history, and CAD score details, which were then compared. Continuous variables are presented as means and standard deviations or medians and interquartile-ranges, and compared via a Kruskal–Wallis test. Categorical data are presented as counts and percentages, and compared using χ^2^-square test. Due to the pilot study character and the main outcome merely being feasibility, no sample size calculation was performed, and a team of experts decided upon 100 patients. Statistical significance was defined by two-tailed *p*-values of <0.05. Data analysis was performed using SPSS 22.0 (IBM, Armonk, NY, USA).

## 3. Results

**Figure 1:**
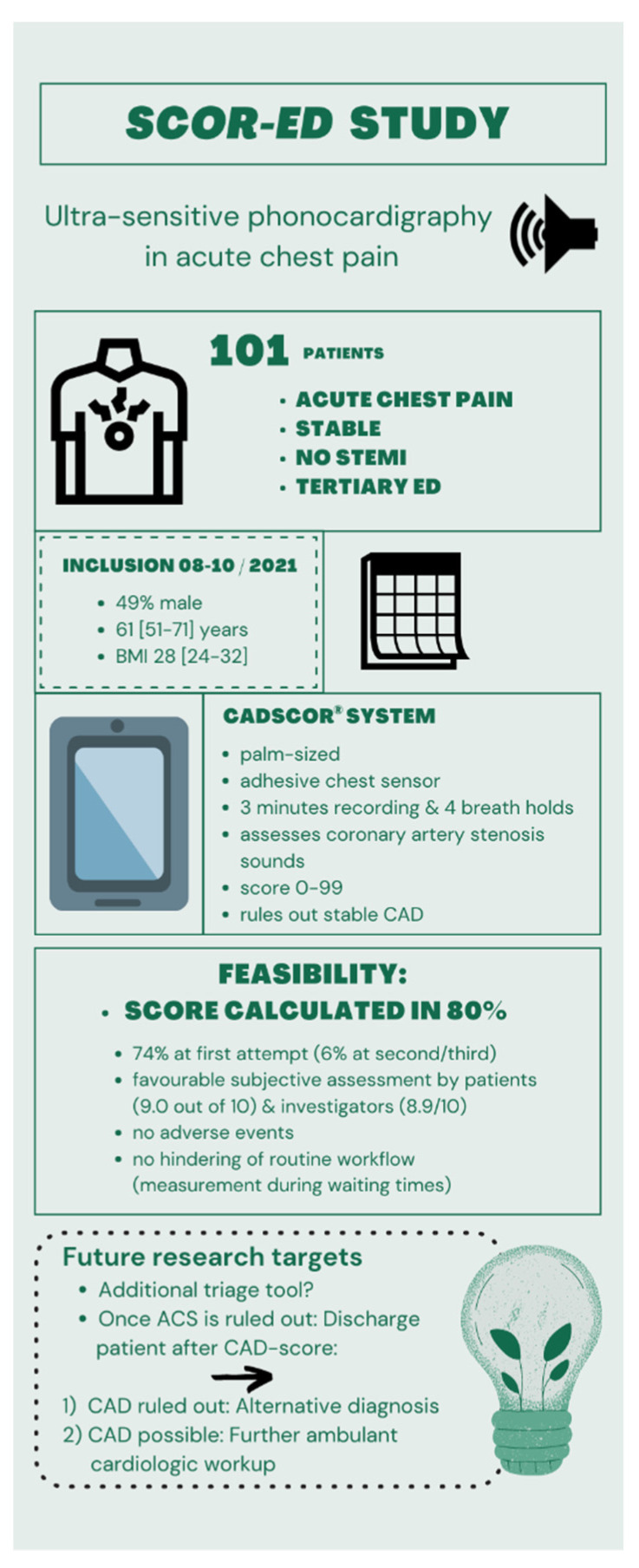
Overview of the main study findings. ED = emergency department; BMI = body mass index; CAD = coronary artery disease; ACS = acute coronary syndrome.

Figure 1 gives an overview of the main study results. A total of 101 patients (49% male; 94% Caucasian; 61 (51–71) years; BMI 28.3 (24.2–31.6)) were included in this study from August to October 2021. No study inclusion hindered or slowed down the patient workflow. No adverse events were recorded. Further data, including details of the current complaints of the whole cohort and stratified into subgroups, are shown in Table 1. In brief, conventionally known associations of gender, previously-known CAD, and well-established risk factors [19] were observed. Table 2 depicts CAD score details and feasibility data. A total of 44% of patients fell into the subgroup of ≤20 points. As age, gender, and hypertension/blood pressure data were integrated in the internal calculation process, they naturally differed in the subgroups.

In 80% of cases, a score was successfully calculated, with 74% at the first, 5% at the second, and 1% at the third attempt. The measurement was stopped by the patient in 9% of cases (e.g., due to incapability to follow necessary breathing commands), and by the investigator in 10% (e.g., due to occurring arrhythmia or tachycardia). In 2% of cases, a measurement was not possible due to technical reasons including inconsistent data analysis by the device (e.g., due to extra systoles or high heart rates) or too weak sound signals.

Feasibility was judged as 9.0 (±1.8) by the patients and 8.9 (±2.6) by the investigators on a 10-point Likert scale (10 being the maximum favorable estimation of feasibility in the current setting of the respective evaluator).

Descriptive laboratory data are found in the Supplementary Table S2.

## 4. Discussion

We demonstrated sufficient feasibility of ultra-sensitive phonocardiography via CAD score in acute chest pain patients at a high-volume ED. Routine workflow was not hindered, no adverse events were noted, and a CAD score was calculated in ¾ of cases at the first attempt. Feasibility was rated high by patients and investigators, and the system suggested a high functionality even under the time- and place-restricting conditions of a busy ED. In times of overcrowded EDs with increasing additional duties, such as comprehensive intensive care, as well as the physical and emotional strain on the staff [13,14,15,16,17], this knowledge seems vital: for instance, the measurements could not have been accepted by the personnel or the patients, time or spatial resources could have not been available, or the busy environment could have prevented sufficient phonocardiographic results. Of note, feasibility was the primary outcome of this study, and not clinical diagnoses or outcomes. To provide data on feasibility was important to build a base for following larger trials.

Further research seems warranted in two major domains:

First, a CAD score should be evaluated as a potential additional triage tool in chest pain patients—not (only) towards discrimination of ACS vs. non-ACS, but rather concerning its potential power to provide additional information on patients’ CAD status right from the start of their evaluation. This especially applies to those patients not being able to provide an estimation on their coronary artery status, or those in whom anamnesis is not sufficiently possible (e.g., language barriers, dementia, etc.). Hints towards a CAD score potentially identifying sicker patients (e.g., lower SpO2, higher cfPWV, or higher NT-proBNP values) should be investigated in future research as well.

Second, and of higher priority, the CAD score performs very well in ruling out stable CAD. Applying this information, alternative diagnoses for the chief complaint of chest pain can become more probable after having already ruled out ACS via the known standard algorithm. Currently, many patients are discharged with provisional diagnoses of their complaints, for instance musculoskeletal thoracic pain or radiating gastric pain [20]. However, with CAD forming a major international health burden with growing incidences [21], ruling out ACS may in the future not be enough. In fact, after having ruled out ACS, rather than discharging patients with an unknown etiology of their symptoms, a further sub-classification into two groups seems thinkable: (1) stable CAD ruled out by CAD score, making a non-cardiac symptom etiology more probable; (2) stable CAD not ruled out by CAD score, making further workup at a cardiologist’s office or a cardiologic outpatient department a logical next step to be advised. With an increasing emphasis on shared decision making, and expensive resources often being consumed with little effect on clinical outcome [22], this cost- and time-effective approach could aid in prioritizing care for individuals who are at particular risk for major adverse cardiac events (MACE). A recently shown prognostic potential of the CAD score towards all-cause mortality and future myocardial infarction emphasizes these thoughts [23].

A study setting with a larger sample size and specially designed to investigate the above-raised points should be aimed for in future research endeavors.

**Key points:**

- Ultra-sensitive phonocardiography in chest pain patients presenting to an emergency department is feasible.
- The CAD score is already utilized to rule out stable coronary artery disease.
- After having ruled out acute coronary syndrome via a guideline-directed algorithm, the CADScor^®^System could help to classify chest pain patients into those with and without coronary artery disease, making a specific further cardiovascular workup possible.

## 5. Conclusions

Ultra-sensitive phonocardiography is feasible in acute chest pain patients presenting to a tertiary emergency department. Thus, a CAD score measured during routine waiting times could potentially serve as an additional tessera in a diagnostic pathway for thoracic pain after having ruled out acute coronary syndrome.

## Figures and Tables

**Table 1:** Patient’s demographics, including chronic conditions and details of their current episode of chest pain. Values are given for the total study cohort and subgroups concerning gender, known coronary artery disease (CAD), calculated CAD score, and final diagnosis regarding acute coronary syndrome (ACS). Categorical data are presented as counts and percentages, continuous data as medians and interquartile ranges (IQRs). Categorical data are analyzed using a test for linear association (Maentel–Haenszel chi-square test), continuous data using Kruskal–Wallis test for testing within the subgroups. BMI = body mass index; SpO2 = oxygen saturation; FiO2 = fraction of inspiratory oxygen; AHTN = arterial hypertension; HLP = hyperlipidaemia; DM = diabetes mellitus; MCI = myocardial infarction; CKI = chronic kidney injury; PAD = peripheral artery disease; baPWV = brachial-ankle pulse-wave velocity; cfPWV = carotid-femoral pulse-wave velocity; AP = angina pectoris; HEART = History, ECG, Age, Risk factors, and Troponin; GRACE = Global Registry of Acute Coronary Events; NYHA = New York Heart Association; ECG = electrocardiogram; IMCU = intermediate care unit.

	Total	Male	Female	*p*-Value	No Known CAD	Previously Known CAD	*p*-Value	CAD Score > 20	CAD Score ≤ 20	*p*-Value	CAD Score Not Calculated	No ACS as Final Diagnosis	ACS as Final Diagnosis
**N** (% of total)	105	51 (48.6)	54 (51.4)		71 (67.6)	34 (32.4)		59 (56.2)	25 (23.8)		21 (20.0)	102 (97.1)	3 (2.9)
Male sex, *n* (%)	51 (48.6)				27 (38.0)	24 (70.6)	**0.020**	34 (57.6)	5 (20.0)	**<0.001**	12 (57.1)	49 (48)	2 (66.7)
Caucasian, *n* (%)	99 (94.3)	48 (94.1)	51 (94.4)	0.943	65 (91.5)	34 (100)	0.081	57 (96.6)	22 (88.0)	0.121	20 (95.2)	96 (94.1)	3 (100)
Age, years (IQR)	61 (51–71)	62 (53–71)	61 (49–71)	0.426	57 (48–68)	69 (62–78)	**<0.001**	68 (58–74)	49 (46–56)	**<0.001**	61 (55–72)	61 (51–71)	66 (53–86)
BMI, kg/m^2^ (IQR)	28 (24–32)	28 (23–32)	29 (25–32)	0.348	28 (24–31)	28 (24–33)	0.627	28 (23–32)	25 (23–30)	0.085	29 (25–32)	28 (24–32)	31 (26–37)
Systolic blood pressure, mmHg (IQR)	144 (129–155)	140 (128–150)	148 (129–160)	0.129	144 (130–155)	142 (123–155)	0.578	149 (140–160)	130 (115–147)	**0.009**	138 (127–155)	142 (128–156)	150 (147–153)
Diastolic blood pressure, mmHg (IQR)	80 (67–87)	81 (71–88)	76 (66.5–84.8)	0.137	80 (69–89)	79 (65–86)	0.231	80 (65–86)	79 (70–91)	0.966	80 (75–89)	80 (67–87)	79 (66–92)
SpO2 (FiO2 0.21), % (IQR)	98 (96–100)	97 (96–99)	98 (97–100)	0.066	98 (97–100)	98 (96–99)	0.184	98 (96–99)	100 (98–100)	**<0.001**	96 (95–98)	98 (96–100)	99 (97–100)
Heart rate, beats/min (IQR)	75 (63–87)	77 (62–89)	74 (64–86)	0.396	75 (64–87)	76 (62–87)	0.873	74 (61–84)	67 (61–82)	0.058	88 (83–96)	76 (63–87)	64 (53–75)
**Comorbidities**													
AHTN, *n* (%)	57 (54.3)	27 (52.9)	30 (55.5)	0.938	27 (38.0)	30 (88.2)	**<0.001**	39 (66.1)	6 (24.0)	**<0.001**	12 (57.1)	55 (53.9)	2 (66.7)
HLP, *n* (%)	34 (32.4)	19 (37.3)	15 (27.8)	0.300	14 (19.7)	20 (58.8)	**<0.001**	22 (37.3)	3 (12.0)	**0.013**	9 (42.9)	33 (32.4)	1 (33.3)
DM II, *n* (%)	26 (24.8)	16 (31.4)	10 (18.5)	0.127	13 (18.3)	13 (38.2)	**0.027**	19 (32.2)	1 (4.0)	**0.060**	6 (28.6)	26 (25.5)	0
CAD previously known, *n* (%)	34 (32.4)	24 (47.1)	10 (18.5)	**0.002**				22 (37.3)	3 (12.0)	**0.013**	9 (42.9)	33 (32.4)	1 (33.3)
Previous MCI, *n* (%)	13 (12.4)	8 (15.7)	5 (9.3)	0.313	0	13 (38.2)	**<0.001**	12 (20.3)	0	0.115	1 (4.8)	12 (11.8)	1 (33.3)
Family history of CAD, *n* (%)	27 (25.7)	9 (17.6)	18 (33.3)	0.085	13 (18.3)	14 (41.2)	**0.007**	15 (25.4)	8 (32.0)	0.450	4 (19.0)	25 (24.5)	2 (66.7)
CKI, *n* (%)	5 (4.8)	2 (4.2)	3 (5.6)	0.686	2 (2.8)	3 (8.8)	0.131	1 (1.7)	2 (8.0)	0.182	2 (9.5)	3 (2.9)	2 (66.7)
PAD, *n* (%)	10 (9.5)	7 (13.7)	3 (5.6)	0.154	2 (2.8)	8 (23.5)	**<0.001**	7 (11.9)	2 (8.0)	0.766	1 (4.8)	9 (8.8)	1 (33.3)
**Vascular status**													
BaPWV, m/s (IQR)	14.0 (12.5–15.8)	13.9 (12.7–15.8)	14.1 (12.3–16.0)	0.653	13.5 (12.4–15.7)	14.2 (12.8–17.8)	0.253	14.8 (12.8–17.2)	12.5 (12.0–13.7)	0.429	14.1 (12.7–15.8)	14.0 (12.5–15.8)	12.8 (12.3–12.9)
cfPWV, m/s (IQR)	9.5 (8.4–11.4)	9.4 (8.6–11.4)	9.7 (8.1–11.6)	0.720	9.3 (8.1–11.1)	10.2 (9.4–12.9)	0.061	10.5 (9.0–12.8)	8.3 (7.8–9.1)	**<0.001**	9.8 (9.4–12.1)	10.3 (7.9–13.0)	8.5 (8.0–9.1)
**Smoking status**													
Never, *n* (%)	43 (41.0)	15 (29.4)	28 (51.9)	**0.019**	34 (47.9)	9 (26.5)	**0.037**	23 (39.0)	14 (56.0)	0.080	6 (28.6)	41 (40.2)	2 (66.7)
Former, *n* (%)	38 (36.2)	24 (47.1)	14 (25.9)	**0.024**	18 (25.4)	20 (58.8)	**<0.001**	23 (39.0)	5 (20.0)	0.054	10 (47.6)	37 (36.3)	1 (33.3)
Active, *n* (%)	24 (22.9)	12 (23.5)	12 (22.2)	0.873	19 (36.8)	5 (14.7)	0.169	12 (22.0)	6 (24.0)	0.876	5 (23.8)	24 (23.5)	0
**Chief complaint**													
Typical AP, *n* (%)	92 (87.6)	44 (86.3)	48 (88.9)	0.684	62 (87.3)	30 (88.2)	0.894	50 (84.7)	23 (92.0)	0.446	19 (90.5)	89 (87.3)	3 (100)
Atypical AP, *n* (%)	6 (5.7)	3 (5.9)	3 (5.6)	0.943	4 (5.6)	2 (5.9)	0.959	5 (8.5%	0	0.158	1 (4.8)	6 (5.9)	0
Non-specific chest pain, *n* (%)	7 (6.7)	4 (7.8)	3 (5.6)	0.639	5 (7.0)	2 (5.9)	0.824	4 (6.8)	2 (8.0)	0.759	1 (4.8)	7 (6.9)	0
**Symptom evaluation**													
Pain worsening with exercise, *n* (%)	34 (32.4)	17 (33.3)	17 (31.5)	0.839	20 (28.2)	14 (41.2)	0.183	17 (28.8)	8 (32.0)	0.963	9 (42.9)	33 (32.4)	1 (33.3)
Cardiac origin of pain assumed by patient, *n* (%)	36 (34.3)	19 (37.3)	17 (31.5)	0.533	22 (31)	14 (41.2%	0.303	19 (32.2)	8 (32.0)	0.783	9 (42.9)	35 (34.3)	1 (33.3)
Pain reproducible with palpation, *n* (%)	21 (20.0)	10 (19.6)	11 (20.4)	0.922	18 (25.4)	3 (8.8)	**0.048**	13 (22.0)	5 (20.0)	0.934	3 (14.3)	20 (19.6)	1 (33.3)
HEART score, points (IQR)	4 (3–6)	5 (3–6)	4 (3–5)	0.155	3 (2–4)	6 (5–7)	**<0.001**	5 (3–6)	3 (2–3.5)	**<0.001**	5 (3–5.5)	4 (3–6)	5 (3.5–5.5)
GRACE score, points (IQR)	79 (59–106)	83 (65–111)	72 (58–93)	**0.042**	68 (53–85)	101 (81–123)	**<0.001**	85 (68–111)	56 (47–63)	**<0.001**	83 (70–107)	78 (59–105)	93 (56–122)
NYHA I, *n* (%)	81 (77.1)	35 (68.6)	46 (85.2)	**0.026**	61 (85.6)	20 (58.8)	**0.004**	44 (74.6)	21 (84.0)	0.398	16 (76.2)	78 (76.5)	3 (100)
NYHA II, *n* (%)	20 (19.0)	13 (25.5)	7 (13.0)	0.112	10 (14.1)	10 (29.4)	0.051	13 (22.0)	4 (16.0)	0.638	3 (14.3)	20 (19.6)	0
NYHA III, *n* (%)	3 (2.9)	3 (5.9)	0	0.073	0	3 (8.8)	0.059	2 (3.4)	0	0.323	1 (4.8)	3 (2.9)	0
NYHA IV, *n* (%)	0	0	0		0	0		0	0		0	0	0
**ECG details**													
ST elevation (not significant), *n* (%)	4 (3.8)	2 (3.9)	2 (3.7)	0.919	1 (1.4)	3 (8.8)	**0.050**	2 (3.4)	0	0.255	2 (9.5)	4 (3.9)	0
ST depression (not significant), *n* (%)	4 (3.8)	1 (2.0)	3 (5.6)	0.357	1 (1.4)	3 (8.8)	**0.050**	3 (5.1)	0	0.331	1 (4.8)	4 (3.9)	0
**Outcome**													
Primarily discharged, *n* (%)	85 (81.0)	38 (74.5)	47 (87)	0.102	64 (90.1)	21 (61.8)	**<0.001**	47 (79.7)	23 (92.0)	0.133	15 (71.4)	85 (83.3)	0
Normal ward admission, *n* (%)	16 (15.2)	12 (23.5)	4 (7.4)	**0.022**	4 (5.6)	12 (35.3)	**<0.001**	9 (15.3)	2 (8.0)	0.249	5 (23.8)	15 (14.7)	1 (33.3)
IMCU admission, *n* (%)	4 (3.8)	1 (2.0)	3 (5.6)	0.336	3 (4.2)	1 (2.9)	0.748	3 (5.1)	0	0.254	1 (4.8)	2 (2.0)	2 (66.7)

**Table 2:** Details of the CAD score measurement. Values are given for the total study cohort and subgroups concerning gender, known coronary artery disease (CAD), calculated CAD score, and final diagnosis regarding acute coronary syndrome (ACS). Categorical data are presented as counts and percentages, continuous data as means and standard deviations (SD) or medians and interquartile ranges (IQRs). Categorical data are analyzed using a test for linear association (Maentel–Haenszel chi-square test), continuous data using Kruskal–Wallis test for testing within the subgroups.

	Total	Male	Female	*p*-Value	No Known CAD	Previously Known CAD	*p*-Value	CAD Score > 20	CAD Score ≤ 20	CAD Score Not Calculated	No ACS as Final Diagnosis	ACS as Final Diagnosis
**N** (% of total)	105	51 (48.6)	54 (51.4)		71 (67.6)	34 (32.4)		59 (56.2)	25 (23.8)	21 (20.0)	102 (97.1)	3 (2.9)
Numerical CAD score, points (IQR)	33 (18–45)	42 (33–55)	21 (12–34)	**<0.001**	26 (14–35)	45 (33–55)	**<0.001**	37 (30–53)	14 (8–18)		32 (18–44)	45 (34–62)
Score successfully calculated, *n* (%)	84 (80.0)	39 (76.5)	45 (83.3)	0.380	59 (83.1)	25 (73.5)	0.251				81 (79.4)	3 (100)
Score failed to calculate, *n* (%)	21 (20.0)	12 (23.5)	9 (16.7)	0.380	12 (16.9)	9 (26.5)	0.251				21 (20.6)	0
Measurement stopped by patient, *n* (%)	9 (8.6)	5 (9.8)	4 (7.4)	0.661	4 (5.6)	5 (14.7)	0.120			9 (42.9)	9 (8.8)	0
Measurement stopped by investigator, *n* (%)	10 (9.5)	7 (13.7)	3 (5.6)	0.154	6 (8.5)	4 (11.8)	0.588			10 (47.6)	10 (9.8)	0
Measurement not possible due to technical reason, *n* (%)	2 (1.9)	0	2 (3.7)	0.165	2 (2.8)	0	0.323			2 (9.5)	2 (2.0)	0
Feasibility judged by patient, points on Likert scale 0–10 (±SD)	9.0 (1.8)	8.9 (2.1)	9 (1.5)	0.414	9.2 (1.3)	8.4 (2.5)	0.200	9.3 (1.5)	9.4 (1.0)	7.5 (2.7)	8.9 (1.8)	9.3 (1.2)
Feasibility judged by investigator, points on Likert scale 0–10 (±SD)	8.9 (2.6)	9 (2.4)	8.8 (2.9)	0.687	9.1 (2.6)	8.5 (2.9)	0.188	9.9 (0.4)	10 (0)	4.8 (3.7)	8.9 (2.7)	10 (0)
1 attempt, *n* (%)	79 (75.2)	36 (70.6)	43 (79.6)	0.283	56 (78.9)	23 (67.7)	0.212	55 (93.2)	23 (92.0)	1 (4.8)	76 (74.5)	3 (100)
2 attempts, *n* (%)	15 (14.3)	9 (17.6)	6 (11.1)	0.339	10 (14.1)	5 (14.7)	0.932	3 (5.1)	2 (8.0)	10 (47.6)	15 (14.7)	0
3 attempts, *n* (%)	7 (6.7)	4 (7.8)	3 (5.6)	0.639	2 (2.8)	5 (14.7)	0.062	1 (1.7)	0	6 (28.5)	7 (6.9)	0
4 attempts, *n* (%)	1 (1.0)	1 (2.0)	0	0.301	0	1 (2.9)	0.147	0	0	1 (4.8)	1 (1.0)	0
>4 attempts, *n* (%)	3 (2.8)	1 (2.0)	2 (3.7)	0.592	3 (4.2)	0	0.224	0	0	3 (14.3)	3 (2.9)	0

## Data Availability

The data underlying this article will be shared on reasonable request to the corresponding author.

## Supplementary Materials

The following supporting information can be downloaded at: https://www.mdpi.com/article/10.3390/jpm12040631/s1. Supplementary Table S1: Overview of the measuring procedure; Supplementary Table S2: Results of the initial venous blood gas analysis and subsequent laboratory tests.
