# Supplementary material for: The Feasibility of Ultra-Sensitive Phonocardiography in Acute Chest Pain Patients of a Tertiary Care Emergency Department (ScorED Feasibility Study)
Source: J Pers Med. 2022 Apr 14;12(4):631. doi: 10.3390/jpm12040631 (PMC9028442; doi:10.3390/jpm12040631)
Supplement: Supplementary file 1 [file jpm-12-00631-s001.zip › Supplementary Table S1.pdf]

Supplementary Table S1. Overview of the measuring procedure. CAD = coronary artery disease. The procedure's time frame is fixed and given by the device/the manufacturer [19].

Phase	Step	Time (seconds)	Action
Resting		>300	Patient lies on bed or stretcher
Recording	Pre-recording	30 + 30	Analyzing the recording conditions
	Breathing (#1)	18	No recording
	Breathing pause (#1)	8	Recording
	Breathing (#2)	18	No recording
	Breathing pause (#2)	8	Recording
	Breathing (#3)	18	No recording
	Breathing pause (#3)	8	Recording
	Breathing (#4)	18	No recording
	Breathing pause (#4)	8	Recording
Results	Post-recording	60–120	Score calculation
Approx. total time (seconds)		524–564	
