# Supplementary material for: The Feasibility of Ultra-Sensitive Phonocardiography in Acute Chest Pain Patients of a Tertiary Care Emergency Department (ScorED Feasibility Study)
Source: J Pers Med. 2022 Apr 14;12(4):631. doi: 10.3390/jpm12040631 (PMC9028442; doi:10.3390/jpm12040631)
Supplement: Supplementary file 1 [file jpm-12-00631-s001.zip › Supplementary Table S2.pdf]

Supplementary Table S2. Results of the initial venous blood gas analysis and subsequent laboratory tests. Values are given for the total study cohort and subgroups concerning gender, known coronary artery disease (CAD), calculated CAD score, and final diagnosis regarding acute coronary syndrome (ACS). Categorical data are presented as counts and percentages, continuous data as medians and IQRs. Categorical data are analyzed using a test for linear association (Maentel–Haenszel chi-square test), continuous data using Kruskal–Wallis test for testing within the subgroups. CO2 = carbon dioxide; HCO3- = bicarbonate; Hct = haematocrit; MCV = mean corpuscular volume; MCHC = mean cell hemoglobin concentration; INR = international normalized ratio; APTT = activated partial thromboplastin time; BUN = blood urea nitrogen; ASAT = aspartate-aminotransferase; ALAT = alanine-aminotransferase; GGT = gamma-glutamyltransferase; LDH = lactic acid dehydrogenase; CK = creatine kinase; NT-proBNP = N-terminal pro brain natriuretic peptide; Hs-TnT = high-sensitive troponin-T; GFR = glomerular filtration rate; CRP = C-reactive protein; VWF = Van-Willebrand factor; ADAMTS13 = a disintegrin and metalloproteinase with a thrombospondin type 1 motif member 13; TSH = thyroid stimulating hormone; FT4 = free thyroxine; FT3 = free triiodothyronine; HDL = high-density lipoprotein; LHL = low-density lipoprotein; LP(a) = lipoprotein(a).

	**Total** *n* = 105	**Male** *n* = 51	**Female** *n* = 54	**No Known CAD** *n* = 71	**Previously Known CAD** *n* = 34	**CAD score > 20** *n* = 59	**CAD score ≤ 20** *n* = 25	**CAD score Not Calculated** *n* = 21	**No ACS as Final Diagnosis** *n* = 102	**ACS as Final Diagnosis** *n* = 3
**Initial venous BGA**										
pH	7.38 (7.36–7.41)	7.38 (7.35–7.41)	7.39 (7.37–7.41)	7.39 (7.36–7.41)	7.37 (7.36–7.4)	7.39 (7.36–7.41)	7.38 (7.35–7.41)	7.38 (7.37–7.42)	7.39 (7.36–7.41)	7.40 (n.a.)
pCO2, mmHg	43(39–48)	44 (40–49)	42 (38–47)	44 (40–49)	41 (38–47)	43 (40–48)	41 (38–52)	44 (40–47)	43 (39–48)	47 (n.a.)
Lactate, mmol/L	1.2 (1.0–1.6)	1.3 (1.1–1.8)	1.1 (0.9–1.5)	1.1 (0.8–1.4)	1.5 (1.1–2)	1.3 (1–1.5)	1.0 (0.7–1.6)	1.5 (1.13–2.4)	1.2 (0.9–1.6)	1.4 (n.a.)
HCO3-, mmol/L	24 (23–25)	24 (23–25)	24 (23–25)	24 (23–25)	23 (22–24)	24 (23–25)	24 (22–25)	24 (23–25)	24 (23–25)	23 (n.a.)
**Serum laboratory values**										
Erythrocytes, G/L	5 (4–5)	5 (4–5)	4 (4–5)	5 (4–5)	5 (4–5)	5 (4–5)	5 (4–5)	5 (4–5)	5 (4–5)	4 (n.a.)
Hemoglobin, g/dL	13 (12–14)	14 (13–15)	13 (12–13)	13 (12–14)	13 (12–15)	13 (12–14)	13 (12–14)	14 (13–15)	13 (12–14)	13 (n.a.)
Hct, %	40 (36–43)	41 (38–45)	39 (36–40)	40 (36–42)	40 (36–44)	39 (35–43)	40 (36–41)	41 (40–44)	40 (36–43)	40 (n.a.)
MCV, fL	89 (86–91)	89 (86–92)	88 (86–91)	88 (86–91)	90 (87–92)	89 (86–91)	87 (85–91)	90 (87–94)	89 (86–91)	89 (n.a.)
MCH, pg	30 (29–31)	30 (29–31)	29 (28–31)	30 (28–31)	30 (29–31)	30 (28–31)	29 (28–30)	30 (29–31)	30 (28–31)	30 (n.a.)
MCHC, g/dL	33 (32–34)	33 (33–34)	33 (32–34)	33 (33–34)	33 (33–34)	33 (33–34)	33 (33–34)	33 (32–34)	33 (33–34)	33 (n.a.)
Platelet, G/L	240 (196–284)	221 (187–263)	250 (216–295)	246 (205–282)	222 (178–296)	238 (194–273)	248 (219–290)	230 (161–314)	240 (193–284)	258 (n.a.)
Leucocyte, G/L	8 (7–10)	9 (7–11)	8 (6–9)	8 (6–9)	8 (7–13)	8 (7–9)	8 (6–9)	10 (7–13)	8 (7–10)	10 (n.a.)
Quick, %	94 (78–104)	89 (77–101)	95 (80–109)	94 (79–106)	92 (78–103)	94 (77–101)	94 (79–107)	92 (83–118)	94 (78–10)	91 (n.a.)
INR	1.0 (1.0–1.1)	1.1 (1.0–1.1)	1.0 (1.0–1.1)	1.0 (1.0–1.1)	1.0 (1.0–1.1)	1.0 (1.0–1.1)	1.0 (1.0–1.1)	1.0 (0.9–1.1)	1 (1–1.1)	1.0 (n.a.)
APTT, sec	37 (34–3)	37 (34–39)	37 (34–40)	37 (35–40)	37 (32–39)	37 (35–39)	36 (35–40)	34 (33–39)	37 (34–39)	37 (n.a.)
Fibrinogen, mg/dL	353 (308–407)	352 (307–434)	358 (309–399)	335 (291–390)	400 (348–470)	361 (316–407)	329 (279–347)	400 (351–576)	355 (309–407)	345 (n.a.)
Sodium, mmol/L	138 (137–140)	138 (137–139)	138 (137–140)	138 (137–140)	137 (136–139)	138 (136–140)	139 (137–140)	138 (136–140)	138 (137–140)	138 (n.a.)
Potassium, mmol/L	4.1 (3.8–4.3)	4.1 (3.9–4.4)	3.9 (3.7–4.2)	3.9 (3.7–4.1)	4.1 (4.0–4.5)	4.1 (3.8–4.4)	3.9 (3.7–4.0)	3.9 (3.7–4.1)	4.0 (3.8–4.3)	4.0 (n.a.)
Chloride, mmol/L	103 (100–105)	103 (101–104)	103 (99–105)	103 (101–105)	103 (100–104)	103 (100–104)	104 (102–106)	101 (99–103)	103 (100–105)	104 (n.a.)

Calcium, mmol/L	2.3 (2.2–2.4)	2.3 (2.2–2.4)	2.3 (2.3–2.4)	2.3 (2.2–2.4)	2.3 (2.2–2.4)	2.3 (2.2–2.4)	2.3 (2.2–2.4)	2.3 (2.3–2.4)	2.3 (2.2–2.4)	2.3 (n.a.)
Magnesium, mmol/L	0.9 (0.8–0.9)	0.9 (0.9–1.0)	0.8 (0.8–0.9)	0.9 (0.8–0.9)	0.9 (0.8–0.9)	0.9 (0.8–0.92)	0.9 (0.8–0.9)	0.9 (0.8–0.9)	0.9 (0.8–0.9)	0.9 (n.a.)
Creatinine, mg/dL	0.9 (0.8–1.1)	1.0 (0.9–1.2)	0.8 (0.7–1.0)	0.8 (0.7–1.0)	1.0 (1.0–1.5)	1.0 (0.8–1.1)	0.7 (0.7–1.0)	1.0 (0.7–1.5)	0.9 (0.8–1.1)	1.0 (n.a.)
BUN, mg/dL	15 (12–19)	16 (12–23)	14 (11–17)	14 (11–17)	18 (14–24)	16 (13–22)	12 (10–15)	14 (11–18)	15 (12–19)	18 (n.a.)
Total protein, g/L	72 (68–75)	73 (67–76)	72 (68–75)	72 (69–75)	71 (66–75)	72 (67–75)	71 (65–75)	73 (72–78)	72 (67–75)	74 (n.a.)
Albumine, g/L	42 (39–45)	42 (39–45)	42 (39–45)	43 (41–45)	50 (38–44)	42 (39–45)	44 (39–45)	43 (39–46)	42 (39–45)	42 (n.a.)
Cholinesterase, kU/L	7.6 (6.2–8.7)	7.9 (6.3–8.8)	7.5 (6.2–8.4)	7.8 (6.2–9.0)	7.1 (6.2–8.2)	7.6 (6.2–8.7)	7.3 (6.1–9.1)	7.6 (6.4–8.3)	7.5 (6.2–8.6)	8.0 (n.a.)
Alkaline phosphate, U/L	72 (62–92)	72 (63–94)	70 (61–92)	69 (61–89)	74 (66–94)	70 (62–89)	68 (53–89)	85 (69–104)	72 (63–92)	61 (n.a.)
ASAT, U/L	22 (19–27)	23 (19–32)	22 (19–25)	22 (19–27)	21 (16–29)	22 (18–27)	22 (20–27)	23 (19–31)	22 (19–28)	21 (n.a.)
ALAT, U/L	21 (17–32)	23 (17–41)	21 (17–26)	22 (18–31)	20 (15–36)	20 (16–30)	22 (18–35)	25 (21–34)	21 (17–33)	18 (n.a.)
GGT, U/L	27 (16–47)	38 (18–67)	20 (16–32)	24 (16–45)	36 (17–72)	31 (18–51)	16 (12–24)	41 (24–60)	27 (16–48)	29 (n.a.)
LDH, U/L	187 (163–218)	183 (156–213)	191 (175–220)	187 (169–218)	194 (158–220)	193 (160–221)	184 (167–216)	181 (160–216)	187 (164–218)	180 (n.a.)
CK, U/L	92 (63–126)	97 (64–155)	89 (59–116)	95 (68–147)	70 (49–107)	93 (62–129)	94 (68–144)	88 (58–105)	93 (63–127)	84 (n.a.)
CKMB, %	16 (11–24)	16 (12–25)	15 (9–23)	15 (9–22)	24 (15–39)	15 (10–24)	22 (14–58)	14 (9–33)	16 (11–24)	n.a.
NT-proBNP, pg/mL	164 (74–470)	132 (53–478)	169 (86–469)	124 (48–207)	405 (141–3133)	224 (113–569)	107 (41–170)	96 (73–595)	157 (73–475)	251 (n.a.)
Hs-TnT 1, ng/L	8 (4–19)	9 (4–28)	5 (4–13)	4 (4–10)	14 (9–32)	10 (5–23)	4 (4–5)	9 (4–18)	8 (4–14)	26 (n.a.)
Hs-TnT 2, ng/L	11 (5–27)	19 (6–35)	10 (4–22)	9 (4–22)	23 (9–33)	14 (7–30)	5 (4–10)	9 (4–35)	10 (5–26)	52 (n.a.)
Hs-TnT 3, ng/L	29 (10–36)	36 (16–114)	18 (6–32)	24 (4–34)	34 (11–89)	24 (10–34)	140 (140–141)	n.a.	23 (9–63)	29 (n.a.)
Glucose, mg/dL	102 (92–118)	102 (92–118)	102 (91–119)	99 (89–110)	107 (99–125)	105 (93–120)	94 (88–105)	102 (95–132)	102 (92–118)	107 (n.a.)
GFR, mL/min/1.73m^2	41 (33–57)	47 (33–57)	35 (32–82)	56 (35–70)	35 (27–48)	43 (34–61)	49 (44–54)	35 (27–54)	38 (33–56)	58 (n.a.)
CRP, mg/dL	0.3 (0.1–0.6)	0.3 (0.1–0.9)	0.3 (0.1–0.5)	0.2 (0.1–0.4)	0.6 (0.1–2.1)	0.3 (0.1–0.6)	0.2 (0.1–0.4)	0.6 (0.2–1.2)	0.3 (0.1–0.7)	0.4 (n.a.)
VWF antigen, %	152 (109–225)	153 (115–219)	144 (106–228)	135 (106–202)	208 (117–247)	149 (103–224)	133 (100–161)	208 (155–273)	151 (108–226)	218 (n.a.)
VWF activity, %	145 (111–200)	144 (110–178)	146 (110–204)	135 (105–174)	174 (118–235)	134 (106–202)	133 (90–167)	178 (149–213)	144 (110–197)	201 (n.a.)
VW-Ristocetin, %	137 (104–206)	136 (105–193)	141 (99–211)	134 (100–163)	171 (105–229)	137 (101–185)	128 (65–151)	175 (135–231)	136 (104–193)	220 (n.a.)
ADAMTS13 activity, %	86 (79–99)	86 (80–96)	86 (78–102)	86 (79–98)	86 (79–106)	86 (80–98)	88 (84–99)	84 (74–110)	86 (79–97)	106 (n.a.)
TSH, µIU/mL	1.2 (0.9–1.9)	1.2 (0.9–2.0)	1.3 (0.8–1.8)	1.3 (0.8–1.9)	1.2 (0.9–2.5)	1.3 (0.9–2.0)	1.2 (1.0–1.7)	1.1 (0.6–2.5)	1.3 (0.9–1.9)	1.0 (n.a.)
FT4, ng/dL	1.2 (1.1–1.5)	1.2 (1.1–1.5)	1.3 (1.1–1.5)	1.2 (1.1–1.5)	1.3 (1.1–1.5)	1.3 (1.1–1.5)	1.2 (1.0–1.3)	1.4 (1.1–1.5)	1.3 (1.1–1.5)	1.2 (n.a.)
FT3, pg/mL	2.9 (2.7–3.2)	3.0 (2.9–3.3)	2.8 (2.6–3.0)	2.9 (2.7–3.2)	2.9 (2.6–3.2)	2.9 (2.7–3.2)	2.8 (2.7–3.1)	3.0 (2.6–3.3)	3.0 (2.67–3.2)	3.2 (n.a.)
Phosphate, mmol/L	1.1 (0.9–1.3)	1.1 (0.9–1.3)	1.1 (1.0–1.3)	1.1 (1.0–1.3)	1.1 (0.9–1.3)	1.1 (1.0–1.3)	1.2 (1.0–1.2)	1.1 (0.9–1.3)	1.1 (1–1.3)	1 (n.a.)
Iron, µg/dL	66 (42–93)	70 (41–98)	65 (45–84)	66 (52–93)	57 (32–88)	66 (40–91)	66 (59–113)	60 (39–82)	66 (41–93)	66 (n.a.)
Transferrin, mg/dL	239 (215–268)	232 (214–264)	244 (217–272)	239 (215–261)	239 (214–270)	233 (214–268)	244 (229–267)	239 (205–279)	239 (215–268)	231 (n.a.)
Transferrin saturation, %	19 (14–27)	21 (14–30)	18 (14–24)	20 (15–29)	16 (10–26)	19 (14–27)	20 (16–31)	17 (12–23)	19 (14–27)	20 (n.a.)
Ferritin, µg/L	133 (60–237)	181 (84–277)	102 (48–180)	113 (523–237)	159 (81–238)	136 (76–212)	72 (45–191)	218 (53–283)	132 (60–237)	152 (n.a.)
Triglycerides, mg/dL	117 (72–176)	120 (69–170)	105 (68–198)	109 (65–184)	123 (78–165)	126 (65–179)	72 (64–141)	109 (95–170)	114 (70–172)	176 (n.a.)
Cholesterol, mg/dL	166 (123–202)	158 (119–192)	186 (150–223)	188 (158–214)	140 (103–163)	165 (115–203)	158 (125–188)	184 (157–190)	173 (123–202)	165 (n.a.)
HDL-cholesterol, mg/dL	47 (39–65)	45 (34–61)	57 (47–69)	52 (45–73)	40 (30–60)	45 (36–65)	59 (42–74)	48 (41–52)	48 (40–66)	29 (n.a.)
Cholesterol/HDL quotient	3.6 (2.5–4.6)	3.4 (2.6–4.7)	3.5 (2.0–4.7)	3.5 (2.5–4.6)	3.4 (2.3–4.5)	3.4 (2.1–5.4)	2.6 (2.0–4.1)	3.5 (3.4–4.6)	3.5 (2.4–4.6)	6 (n.a.)
LDL-cholesterol, mg/dL	92 (53–120)	80 (52–118)	106 (62–131)	113 (68–123)	66 (40–99)	92 (42–119)	68 (53–112)	120 (77–123)	90 (52–121)	101 (n.a.)
LP(a), nmol/L	28 (12–156)	56 (12–159)	22 (7–154)	23 (8–124)	101 (16–193)	42 (14–131)	16 (9–193)	74 (8–192)	27 (11–148)	159 (n.a.)
